# Rehabilitation for children with chronic acquired brain injury in the Child in Context Intervention (CICI) study: study protocol for a randomized controlled trial

**DOI:** 10.1186/s13063-022-06048-8

**Published:** 2022-02-22

**Authors:** Nina Rohrer-Baumgartner, Ingvil Laberg Holthe, Edel Jannecke Svendsen, Cecilie Røe, Jens Egeland, Ida M. H. Borgen, Solveig L. Hauger, Marit V. Forslund, Cathrine Brunborg, Hege Prag Øra, Hilde Margrete Dahl, Line Kildal Bragstad, Eli Marie Killi, Maria Sandhaug, Ingerid Kleffelgård, Anine Pernille Strand-Saugnes, Ingeborg Dahl-Hilstad, Jennie Ponsford, Laraine Winter, Shari Wade, Marianne Løvstad

**Affiliations:** 1 Department of Research, Sunnaas Rehabilitation Hospital, Nesodden, Norway; 2 Institute of Psychology, University of Oslo, Oslo, Norway; 3 Department of Nursing and Health Promotion, Oslo Metropolitan University, Oslo, Norway; 4 Research Center for Habilitation and Rehabilitation Services and Models (CHARM), Institute of Health and Society, University of Oslo, Oslo, Norway; 5 Department of Physical Medicine and Rehabilitation, Oslo University Hospital, Oslo, Norway; 6 Institute of Clinical Medicine, University of Oslo, Oslo, Norway; 7 Vestfold Hospital Trust, Tønsberg, Norway; 8 Oslo Centre for Biostatistics and Epidemiology, Research Support Services, Oslo University Hospital, Oslo, Norway; 9 Department of Clinical Neurosciences for Children, Oslo University Hospital, Oslo, Norway; 10 Department of Occupational Therapy, Prosthetics and Orthotics Oslo Metropolitan University, Oslo, Norway; 11 Statped: Norwegian Service for Special Needs Education, Oslo, Norway; 12 The Norwegian Association for the Traumatically Injured, Oslo, Norway; 13 School of Psychological Sciences, Monash University, Melbourne, Australia; 14 Monash-Epworth Rehabilitation Research Centre, Richmond, Australia; 15 Philadelphia Research and Education Foundation, Philadelphia, PA USA; 16 Nursing Service, Department of Veterans Affairs Medical Center, Philadelphia, PA USA; 17 Division of Physical Medicine and Rehabilitation, Cincinnati Children’s Hospital Medical Center, Cincinnati, OH USA; 18 University of Cincinnati College of Medicine, Cincinnati, OH USA

**Keywords:** Pediatric brain injury, Acquired brain injury, Goal-oriented, Family intervention, School, Community, Health care needs, Parenting, Online treatment, Telerehabilitation

## Abstract

**Background:**

Pediatric acquired brain injury (pABI) is associated with long-term cognitive, behavioral, social, and emotional problems, which may affect the quality of life, school, and family functioning. Yet, there is a lack of evidence-based community-centered rehabilitation programs for chronic pABI and these children do not systematically receive comprehensive rehabilitation. The Child In Context Intervention (CICI) study is a pragmatic randomized controlled trial (RCT) for children with chronic pABI, which aims to evaluate the effectiveness of an individualized and goal-oriented intervention targeting everyday functioning of the child and family.

**Methods:**

Children aged 6–16 years with MRI/CT-verified intracranial abnormalities will be included in the CICI study if they have persistent self- or parent-reported cognitive, emotional, and/or behavioral challenges 1 year or more after insult and attend school regularly. A total of 70 families will be randomized 1:1 to an intervention or a control group. The intervention consists of seven family sessions, one parent seminar, and four school sessions delivered over approximately 6 months. The parent seminar will be held in person, and the other sessions will mainly be video based. The children’s and families’ self-reported major challenges in everyday life will be targeted using SMART goals. Evidence-based strategies, when available, will be applied to achieve the goals, combined with psychoeducation. Goal attainment scaling (GAS) will be used to evaluate goal attainment. Data is collected at baseline and after approximately 6 and 9 months. External assessors are blinded to group allocation. Primary outcomes are parent-reported brain injury symptoms in children and parenting self-efficacy at 9 months of follow-up. Secondary outcomes include child-reported brain injury symptoms, quality of life, executive functioning in daily life, parent emotional symptoms, family functioning, and unmet family health care needs. A process evaluation will be conducted.

**Discussion:**

The current study provides an innovative approach to rehabilitation for children in the chronic phase of ABI and their families. This complex intervention may contribute to the development of evidence-based, high-quality rehabilitation for a large patient group, which is underrepresented in clinical research. It may also improve collaboration between specialized rehabilitation facilities, schools, and local health care services. Inclusion for the trial started in April 2021.

**Trial registration:**

ClinicalTrials.govNCT04798859. Registered on March 15, 2021

**Supplementary Information:**

The online version contains supplementary material available at 10.1186/s13063-022-06048-8.

## Introduction

### Background

Acquired brain injury (ABI) is inflicted after birth due to traumatic injury (e.g., falls, sports and traffic accidents, or assault) or non-traumatic insult (such as stroke, encephalitis, tumor, or hypoxia) and is the single most common cause of pediatric mortality and morbidity [1, 2]. Survivors frequently struggle with chronic cognitive, behavioral, social, and emotional problems, resulting in reduced everyday functioning and quality of life [3–5]. Pediatric ABI (pABI) often alters the pace and course of central nervous system maturation, affecting the whole life-course of the children involved [6, 7]. Thus, skills may fail to develop normally and challenges may become increasingly visible over time as societal demands and expectations increase [3, 6, 8]. Despite potentially severe long-term consequences, children with ABI do not currently receive systematic comprehensive rehabilitation in the chronic stage (more than 1 year after insult), resulting in unmet health care needs [3, 9–13] and placing them at risk for poor health outcomes and decreased quality of life. The lack of rehabilitation in the chronic stage leaves us with the paradox that symptom management for those whose lives are the most affected by the insult — children — is largely left to people without training in brain injury rehabilitation — their parents or caregivers. This is particularly unfortunate as having a child with ABI can negatively impact family functioning and parental mental health, which are in turn important predictors for the child’s functioning and well-being [14–17].

Similarly, teachers are left to manage the symptoms of pABI in the school setting, despite a lack of knowledge about pABI in schools [18, 19]. Alarmingly, many teachers of children with ABI may not even be aware that one of their students has suffered a brain injury/insult [20, 21]. A lack of collaboration between the health care and educational system [13, 22] may contribute to this. As a result, children and adolescents with ABI are under-identified for educational services and those who receive educational services are often under-served [23, 24]. To make matters worse, brain injuries in children tend to be “forgotten” over time in the educational system [25], even though many of these children and adolescents have persistent impairments and are in need of long-term follow-up [13, 23, 25, 26].

There is no solid volume of evidence-based treatment recommendations for cognitive rehabilitation after pABI, as research on pediatric brain injury rehabilitation is scarce. However, indirect interventions that aim to establish compensatory strategies and environmental adaptations to improve the children’s functional performance are recommended, while the sole use of so-called direct rehabilitation interventions, which focus on retraining of cognitive and functional impairment, holds less promise in the chronic stage [8, 27–35]. The few randomized controlled trials (RCTs) testing post-pABI parenting/family interventions indicate that problem-solving interventions are effective in reducing family conflict, enhancing parental functioning, reducing parental stress and psychological problems, and reducing internalizing and externalizing behavior in children and adolescents with ABI [36–41]. Despite the consensus that the complex interplay between brain injury symptoms, family function, and the child’s environment necessitates inclusion of the child’s family and school [29, 42], we are not aware of any RCTs that target both of these important environments in interventions directed towards chronic pABI. A recent evidence-based description of general rehabilitation describes effective rehabilitation as a complex, person-centered process with a range of activities, including but not limited to task practice, education, and psychosocial support, delivered by an expert multidisciplinary team working within the biopsychosocial model of illness [43]. Furthermore, as these children live with a wide variety of pABI-related difficulties, effective rehabilitation needs to be flexible and tailored to the individual’s needs, monitored, and modified if needed [43]. Brain injury interventions which fulfill these requirements have until now only targeted the adult population [44, 45], with research on pABI lagging behind.

The use of telerehabilitation, the delivery of rehabilitation services via information and communication technologies, expands the access to rehabilitation services and allows a higher frequency of rehabilitation services due to reduced cost and travel time [46] and thus holds promise for patients with pABI. In fact, it may be particularly suitable for patients with ABI as these are often severely affected by fatigue, which makes travel particularly burdensome. Furthermore, it offers a chance to increase the ecological validity of rehabilitation. Sometimes, maladaptive behavior or emotional reactions may be intense and highly prevalent in the home environment but hard to observe in the clinic. By allowing a more direct access to the home environment, telerehabilitation may be particularly suitable when tailoring interventions to everyday life, compared to rehabilitation delivered in a hospital setting. An effective way of doing so is by identifying and working towards individual rehabilitation goals for the child and parents, which ensures the family’s place at the helm of decision-making and increases their focus on the specific rehabilitation strategies [47, 48]. Involving parents in goal-oriented interventions has yielded promising results [8] and enhances adherence to the treatment program and the feeling of relevance that the treatment holds for the family [49].

The current study is modeled after two RCTs for adults with TBI [44, 45] and has been adapted to the pediatric brain injury population with a complex and innovative design to ensure a holistic approach.

### Objectives and hypotheses

The main aim of the Child in Context Intervention (CICI) is to enhance everyday functioning of children with ABI in the chronic stage in the home and school environment and to improve family life. We expect that the intervention, compared to treatment as usual (TAU), will result in reduced parent-reported brain injury symptom severity in children (H1) and improved parental self-efficacy (H2). We furthermore expect that the intervention will result in lower levels of child-reported brain injury symptoms (H3), fewer unmet health care needs in the families (H4), and improved everyday executive functioning (H5) and quality of life for children (H6). Improved family functioning (H7), parental mental health (H8), and finally, reduced child- and parent-reported severity of the individually defined target outcome areas (main pABI-related problems in daily life) (H9) are also expected. Group differences will be evaluated approximately 6 months and 9 months after baseline, with 9 months (T3) being the primary endpoint. In the treatment group, we expect to see positive goal attainment (H10), and high satisfaction of the participating stakeholders (children, parents, teachers, and rehabilitation professionals) with the intervention program (H11).

## Methods

### Study design

**Fig. 1:**
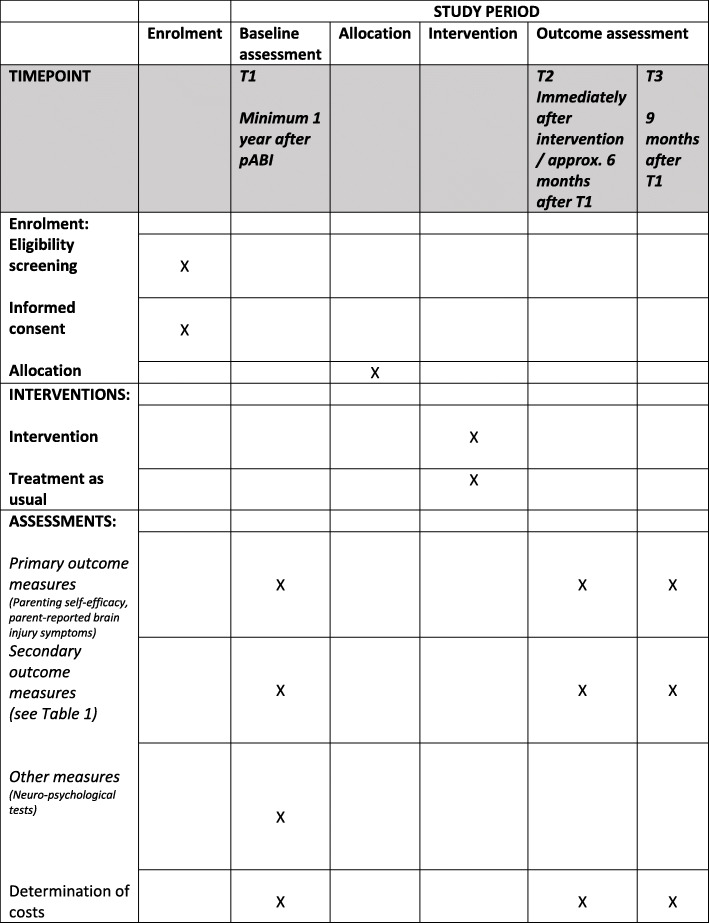
Standard Protocol Items: Recommendations for Interventional Trials (SPIRIT)

The present study is a two-group RCT with a mixed-methods design. A total of 70 children and their families will be randomly assigned to the CICI intervention or control group. The intervention team includes the child, parents (in an assumed minority of cases only one parent), the child’s teacher, a therapist, and a special education specialist. These will work in close collaboration towards the goals of the participating child and family. Most sessions, except for the parent seminar, will be delivered by videoconference, thus reducing the burden of travel and time for the families and therapists. Assessment will be performed at baseline (T1), approximately 6 months later (T2), and 9 months after baseline (T3). The total duration of participation is 9 months. The day of the baseline assessment is regarded as the time point for inclusion. When preparing this protocol, we used the SPIRIT reporting guidelines [50] and a figure with the standard protocol items (SPIRIT) is presented in Fig. 1. The SPIRIT checklist is available as an additional file (Additional file 1).

Potentially eligible families will be invited by letter and screened for inclusion by phone, following an established screening protocol. All three therapists conduct the screening, as it is not feasible for one person to follow up all the phone calls. The baseline assessment (T1) will include an interview with at least one parent including anamnestic information and current use of healthcare services, a brief cognitive screening of the child with the Similarities and Matrix subtests from the Wechsler Intelligence Scale for Children, Fifth edition (WISC-V) [51] for estimating intellectual ability and questionnaires for assessing (1) executive functions of the child in daily life [52], (2) health-related quality of life of the child and brain injury symptoms [53, 54], (3) emotional symptoms of the parents [55, 56], (4) parenting self-efficacy [57], (5) family functioning [58], and (6) unmet health care needs [59] (Table 1). The baseline assessment will also include identification of three main problems in daily life, related to the child’s ABI. The main problems in daily life will be reported by children, according to their age and cognitive functioning, and their caregivers, and rated individually on a 5-point Likert scale according to the level of their burden. The families in the intervention group will rate how helpful they expect the intervention to be on a scale from 1 to 10 during sessions 1 and 3. The intervention was developed according to the UK Medical Research Council framework [60] and also complies with the updated framework, as the CICI study strives to be a solution for real-world practice [61]. The CICI study will be carried out in close collaboration between the specialized health care system, Statped (the Norwegian Service for Special Needs Education), teachers, and local rehabilitation services. Senior scientists will oversee 10% of family sessions to ensure adherence to protocol. Due to the small sample size and as we are documenting dates and times of the intervention, an external data protocol committee is not judged to be necessary.

**Table 1:** Outcome measures, respondents, and time points

Outcome measure topic	Name of measure	Respondents	Time points
Brain injury symptoms	Health and Behavior Inventory (HBI) [54]	Parents, children from 7 years	T1, T2, T3
Parenting self-efficacy	Tool to measure Parenting Self-Efficacy (TOPSE)^1^ [57]	Parents	T1, T2, T3
Executive functioning at home and in school	Behavior Rating Inventory of Executive Function 2 (BRIEF-2) [52]	Parents, teachers, children from 11 years	T1, T2, T3
Children’s quality of life	The Pediatric Quality of Life Inventory (PedsQL)^1^ [53]	Parents, all children	T1, T2, T3
Unmet healthcare needs of the family	Family Needs Questionnaire (FNQ-P) [59]	Parents	T1, T2, T3
Parents’ symptoms of depression	A brief depression severity measure (PHQ-9) [55]	Parents	T1, T2, T3
Parents’ symptoms of generalized anxiety	A brief measure for assessing generalized anxiety disorder (GAD-7) [56]	Parents	T1, T2, T3
Main ABI-related problems in daily life	Likert scale 1–4	Parents, all children	T1, T2, T3
Participants’ evaluation of the intervention	CICI Evaluation (custom made for the CICI study)	Parents, all children, teachers, therapists	T2
Goal attainment scaling	The goal stairs 1–5 (custom made for the CICI study)	Families with therapists	T2

^1^Different versions are applied for different age groups

A feasibility study, using qualitative and quantitative methods, was applied to evaluate inclusion criteria, feasibility of the intervention, the technical solution, adherence to the study protocol, the acceptability of the intervention to children, parents and therapists, mechanism of action, and outcome measures [62]. The current manuscript describes the final study protocol after feasibility testing.

### Study setting

Baseline assessments will be conducted at Sunnaas Rehabilitation Hospital (Bjørnemyrveien 11, 1453 Bjørnemyr, Norway), which is one of the largest rehabilitation hospitals in Northern Europe and the owner of the present study. Intervention sessions will primarily be delivered by videoconference, while the parent seminar will be held at Sunnaas Rehabilitation Hospital or a conference center in the same region. The study is conducted in close collaboration with Oslo University Hospital and Statped, the Norwegian Service for Special Needs Education.

### Participants

The study population will consist of children with ABI with persistent self- or parentally reported insult-related cognitive, emotional, behavioral, and/or social challenges.

*Inclusion criteria are:*

- School-aged (6–16 years) children with clinical ABI diagnosis and CT/MRI-verified insult-related intracranial abnormalities or loss of consciousness post-insult and verified neurological symptoms in cases where MRI could not be administered. We anticipate to include children with TBI, cerebrovascular incidents, anoxia, encephalitis, brain tumors, and brain injury caused by radiation.
- Time since insult at least 1 year.
- The children report ABI-related cognitive, emotional, behavioral, and/or social problems that affect everyday functioning and/or participation in activities with family, friends, school, and/or community, and/or the parents report these symptoms on behalf of the children.
- The children attend school regularly, with or without insult-related adaptations.
- The family is able to actively participate in goal-oriented work, including having Internet access and speaking sufficient Norwegian. An exception can be made for parents who speak English but are able to understand and read study-related information in Norwegian.

*Exclusion criteria:*

- Children with severe pre- or comorbid neurological or neuropsychiatric disorders that would confound assessment and/or treatment outcomes.
- Children with brain tumors or cancer in active treatment or at great risk of relapse (unstable condition).
- Children with severe psychiatric illness or injuries so severe that they are in institutionalized care most of the time.
- Parental severe psychiatric illness, drug abuse, or indications of a history of or risk of domestic violence.

### Outcome measures

The primary outcome measures assess parent-rated severity of children’s brain injury symptoms (Health and Behavior Inventory, HBI) [54] and parenting self-efficacy (Tool to measure Parenting Self-Efficacy, TOPSE) [57] at T3. See Table 1 for an overview of all assessment measures, respondents, and time points.

### Randomization and allocation concealment

Participants will be randomly allocated in a 1:1 ratio to either the intervention group or the control group after completing the baseline (T1) assessment. A Web-based block randomization will be generated by an independent statistician prior to trial start-up to ensure randomization and complete allocation concealment. The allocation will be performed in Viedoc™ (Viedoc Technologies AB, Uppsala, Sweden), the electronic data capture system used in this study (see below). The allocation sequence can be accessed by neither the therapists nor the outcome assessors, only by the study principal investigator. Randomly generated block sizes will be applied. Outcome assessors blinded to group allocation will interview participants and collect information on target outcome areas (main pABI-related problems in daily life) at T2 and T3. For apparent reasons, blinding of participants and interventionists is not possible, but researchers will be blinded to group allocation in the final database, as the participants will be provided with new ID numbers.

### Study interventions

#### Intervention layout

**Fig. 2:**
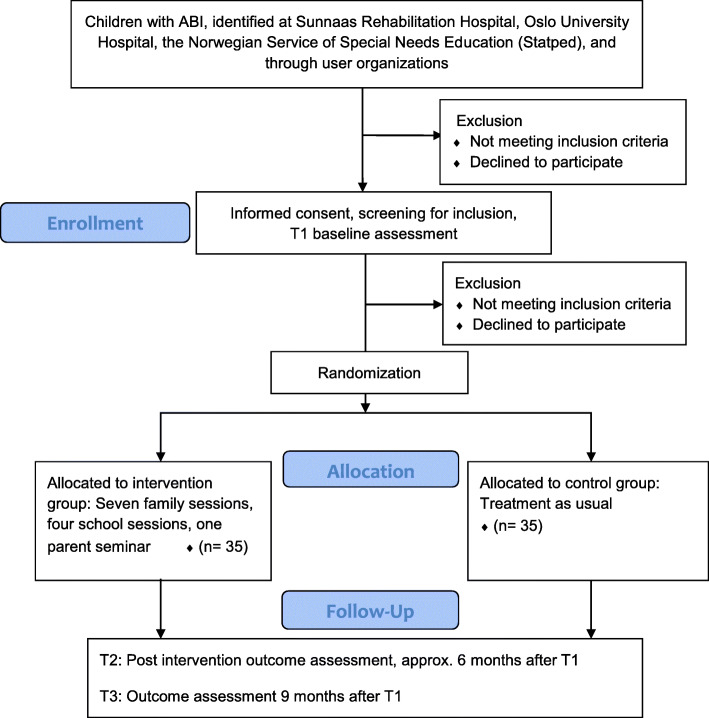
Flowchart of the CICI study

The intervention is inspired by a Norwegian community-based intervention study for adults in the chronic stage of TBI [44], which in turn was modeled after a study by Winter and colleagues [45]. Several alterations were made to adapt the intervention to children and families, e.g., including schools and Statped (the Norwegian Service for Special Needs Education) in establishing and following up school-related strategies and including a parent seminar. A flowchart of the study is presented in Fig. 2.

The intervention lasts for approximately 6 months and consists of 12 sessions, whereof most will be videoconferences, with the possibility of one to two home sessions. Seven 1.5- to 2-h family sessions make up the largest part of the intervention. They will be delivered approximately every 2 to 3 weeks. During the intervention, parents will participate in a 1-day seminar with psychoeducation and experience sharing. For practical reasons, parent seminars will be held in small groups and on fixed dates. One classroom observation will be carried out by a special needs educator before the first of four school sessions, which will be held as three videoconferences and one phone call during the same 6 months as the family sessions and the parent seminar. School sessions will be delivered with longer intervals between family sessions. The last family session marks the end of the intervention.

**Fig. 3:**
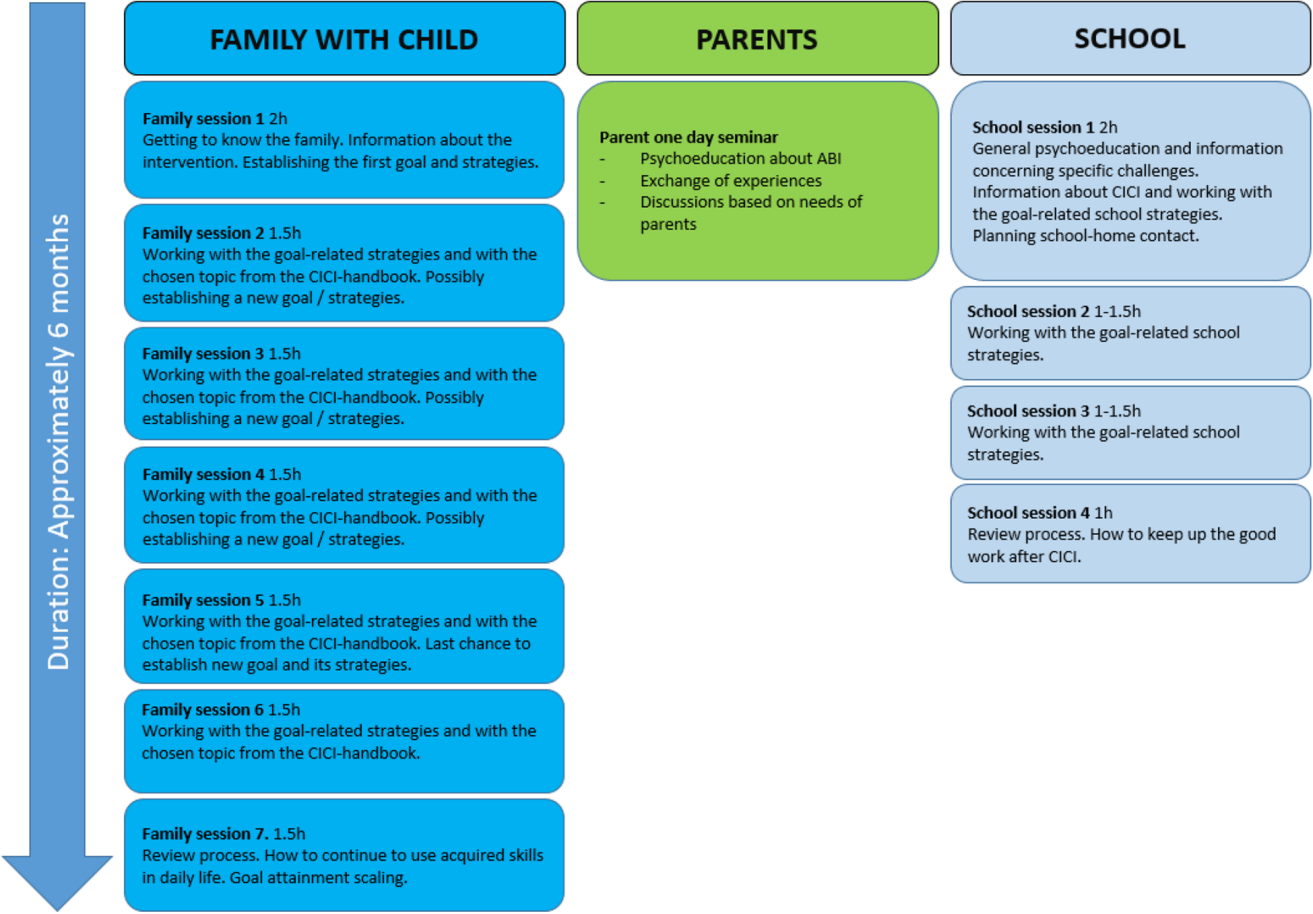
Overview of the intervention sessions

School and family sessions might be conducted in person to accommodate the family’s specific needs, if feasible according to local COVID-19 restrictions and geographical location. Figure 3 provides an overview of the intervention.

### Intervention content

#### Family sessions

Families in the intervention group will, in collaboration with their therapist, establish approximately one to five SMART goals during family sessions. SMART goals will be a main focus in the family sessions and will often, but not always, be based on the target outcome areas (the family’s main pABI-related problems in daily life), depending on the wishes and needs of the family. SMART goals are Specific, Measurable, Achievable, Realistic/Relevant, and Timed [63]. For a quantifiable measure at the end of the intervention (family session 7), goal attainment scaling (GAS) [64] will be applied during goal setting. Current recommendations for the use of GAS will be followed [65]; however, for motivational purposes for the children, a scaling from 1 to 5 will be presented, instead of −2 to 2. Appropriate treatment strategies will be discussed and defined for every SMART goal. Families will be encouraged to actively apply the strategies in daily life during the intervention period. Experiences with the strategies will be discussed during sessions and strategies revised when necessary, but the goals and GAS will not be changed after they have been defined. As the literature on treatment of ABI-related challenges in children in the chronic phase is scarce [6], the intervention strategies will rely on the available evidence-based recommendations existing for the pediatric population [8, 27–30] as well as the evidence-based recommendations given to the adult population, with age-appropriate adaptations (i.e., Translating Evidence-Based Recommendations into Practice [66]). In addition, therapists will draw from the literature on children with neurodevelopmental disorders and typically developing children when choosing rehabilitation strategies, as suggested by Slomine and colleagues [5]. A psychoeducational booklet with a biopsychosocial framework, authored by therapists with long experience from working with children with ABI, will be provided to all participants in the intervention group, including teachers. The topics for the booklet were selected based on recent research about the needs of children with ABI [47] and relevant topics for parents were added. Users contributed feedback during the development of the booklet. The booklet includes the following chapters with information, advice, and links to useful Internet sites: common sequelae after pABI, cognitive difficulties, emotion regulation, social functioning, fatigue, sleep, pain management, psychological well-being, stress management, communication, parenting a child with ABI, and identity after pABI. Relevant topics from the booklet will be discussed during sessions. Children contribute to the extent they are able to. Care is taken to include the children in the goal-related work as much as their age and cognitive skills allow, to motivate them to share their experiences with the strategies and to take part in adapting strategies that do not work as well as intended. No concomitant care or treatment is prohibited or will be retracted during the trial.

#### School sessions

While the SMART goals are defined by the families in collaboration with the therapist, relevant school-related strategies to reach the goals will also be defined, closely monitored and, when necessary, revised in school sessions. Thus, schools will be expected to apply the established goal strategies during the intervention period. For instance, the goal for a child with fatigue to reduce tantrums at home cannot be solved by the family alone, but also requires that the school and teachers understand fatigue and provide an environment where the child can learn and interact with peers without exhausting himself/herself. As an example, appropriate school strategies for fatigue may include noise reduction in the classroom, extra breaks for the child during the day, extra time to complete tasks, and information to classmates to enhance understanding and reduce hurtful comments. In addition to working with the strategies, teachers will receive psychoeducation about pediatric brain injury within a biopsychosocial framework. Parents will be encouraged to participate in school sessions to optimize home-school collaboration. Children are welcome to participate in school sessions if they wish to. Primary health care providers will be invited to attend school sessions when relevant.

#### Parent seminar

In line with recent studies that highlight the importance of including the children’s caregivers in the rehabilitation process [6], and the need to discuss certain topics without children present, a day-long parent seminar targeting family functioning and parenting will be delivered. Topics for the parent seminar include parents’ experiences from working with SMART goals and strategies; parenting after pABI with a particular focus on communication; changes in family dynamics and caring for siblings after pABI; emotional reactions of the family after pABI with a particular focus on feelings of guilt, grief, and embarrassment; and self-care. The topics will be followed up in family sessions if relevant to the goals set. The parent seminar is inspired by interventions carried out in studies recommended in several Cochrane reviews [38, 67–72]. To heighten user involvement, a member of the user organization who has a child with ABI will be invited to participate in parent seminars.

#### Collaboration with local health care professionals

When families wish to invite health care professionals in the municipality with whom they already have established contact to collaborate in the study, these will be asked to participate in some of the intervention sessions, depending on the goals set. Involvement of local resources, including schools, will ensure knowledge transfer from specialized health care to the community. For families who lack help from local health care professionals, the therapists will refer the child to a specialist or support the family in seeking relevant help if judged beneficial. The CICI study will keep track of participation of health care professionals in CICI sessions.

#### Therapists in the CICI study

Three experienced therapists, two neuropsychologists and one pediatric nurse, will deliver the intervention, in close collaboration with an experienced special needs educator who will be in charge of the school sessions. To ensure equal quality of interventions, the three therapists will collaborate closely and frequently discuss SMART goals, goal attainment scaling, and relevant strategies for the goals with each other and with senior researchers. The research group consists of experienced health personnel, and the study has established procedures to deal with unexpected adverse events or medical/psychological issues in acute need of treatment. In such cases, participants will be referred accordingly.

#### Treatment as usual

Children in the control group will receive their usual health care and rehabilitation services (TAU). In Norway, health care in the chronic phase of ABI is largely provided by the municipalities, in cooperation with the specialist health care system. As the provided rehabilitation varies greatly depending on the needs of the children and on the municipalities involved, parents will be interviewed at all time points about the type and amount of health care services provided for each child participant in both groups. This will enable a comparison of service provision between the groups regarding frequency, duration, and profession of the health care professionals involved.

#### Technical solutions and data management

The videoconference solution is provided by the Norwegian Health Net (join.nhn.no) and is delivered by Pexip. It is encrypted and pin codes are used to access virtual meeting rooms, which are locked after all participants have joined. Therapists will use their work computers with an integrated camera and participants use their own computer or tablet with an integrated camera. Laptops can be lent to participants if they do not have a technical device that is suited for videoconferences. To optimize sound, therapists will use USB speakerphones and will lend speakerphones to participants if the sound is judged suboptimal during session 1. The videoconference solution is risk assessed and approved for clinical use by Sunnaas Rehabilitation Hospital. Procedures for clinical use are developed and therapists received training prior to the intervention.

Questionnaires will be sent to participants using Viedoc, a secure electronic data catching system developed for clinical trials. Its homepage cannot be googled but must be accessed with the correct Web address. Every participant will get his/her own username and password to use on their own smartphone, tablet, or computer. The questionnaires will be available in Viedoc for a limited amount of time for each time point and every participant can complete a questionnaire only once. Questionnaires are not visible for people who are not logged in and cannot be printed from Viedoc.

To ensure that the data set is as complete as possible, the therapists will in turn be assigned administrative responsibility of follow-ups of all participants and a scientist will be hired to assist.

Deidentified quantitative data will be stored in Viedoc while recruitment is ongoing and the database on the research server at Sunnaas Rehabilitation Hospital during the latest stage of the study. The qualitative data (the audio recordings of the interviews) will be stored in controlled access folders on the same research server. Tapes and transcripts will be kept locked at Sunnaas Rehabilitation Hospital. All data will be securely contained for 5 years after the end of the project, on the electronic research server at Sunnaas rehabilitation hospital, with restricted access limited to the research group. Deidentified individual clinical trial participant-level data (IPD) will not be shared publicly due to privacy regulations. Important protocol modifications will be reported to the Norwegian Regional Committee for Medical and Health Research Ethics and amendments will be made to the trial registry (Clinicaltrials.gov).

#### Statistical analyses

Descriptive statistics will be used to depict demographics, insult characteristics, and service delivery at baseline as well as acceptability in the intervention group.

Data will be analyzed with linear mixed models taking into account the repeated measures design of the study. All assessment points will be included in the analyses. The main effect of time, treatment, and the interaction between time and treatment will be assessed. The main endpoint for the primary outcome is at T3 (9-month follow-up). The analysis of primary interest in establishing treatment efficacy is a time × group interaction in the direction of the intervention group improving above the levels of the control group at T3. Estimates of mean between-group changes from T1 to T2 and T2 to T3 will also be provided. For primary outcomes, an alpha level of *p* < 0.025 will be applied, as there are two primary outcome measures. A mixed model analysis uses all the available data to compensate for the missing data points. Thus, imputation techniques are not necessary.

Additional analyses including subgroup analyses will be performed, e.g., to compare the effect of the intervention on parent- and child-reported outcomes and outcomes reported by different caregivers (e.g., mothers and fathers). *T*-test analyses will be used for between-group mean comparisons for normally distributed continuous data, and Mann-Whitney *U*-tests for skewed data. Kruskal-Wallis *H* will be used when more than two groups are compared. Individual and treatment-related predictors for goal attainment will be assessed by multivariable regression analysis in the intervention group.

An intention to treat model will be followed, using data from all randomized participants, regardless of whether they complete the intervention or not.

#### Sample size and power calculations

As the precise prevalence and incidence of pABI in Norway is not known, we have established eligibility estimates based on Norwegian and international research regarding the largest subgroups to estimate. In the greater Oslo region (South-Eastern health region), an estimated 105 children per year acquire a brain injury or insult: approximately 35 acquire a TBI (mild TBI not verified by CT or MRI excluded) [73, 74] approximately 22 suffer from cancer in the central nervous system [75], approximately 48 children receive a diagnosis of encephalitis [76, 77], and approximately 18 children experience a stroke each year (perinatal stroke excluded) [78]. About one-third of the surviving children are expected to experience unmet health care needs more than 1 year after injury/insult [79, 80]. Thus, with a rough estimate of 30 eligible children per year in the Oslo region, recruitment for 2 years, including recruitment of children whose injury/insult happened during the last 15 years, the number of eligible families should be sufficient to allow recruitment of 70 families. With an estimated attrition rate of 5–10%, based on our earlier TBI research, around 64 participants are expected to complete all outcome assessments, with 32 children in each intervention arm. Children will be recruited from Sunnaas Rehabilitation Hospital, Oslo University Hospital, Statped (the Norwegian Service for Special Needs Education), and through user organizations.

Effect size and power calculations were conducted using G*Power [81]. Since there are two primary outcomes, the calculation was conducted with an alpha level of 0.025. Two-sided *t*-tests were used as the basis for the analysis. With a statistical power of 0.8 and a sample size 70 (expected *N* = 64 after attrition), we will have an 80% chance of detecting treatment effects of 0.8 (Cohen’s *d*). Results from the feasibility study indicated the measures to be sensitive to the CICI intervention, and a recent RCT [82] which studied the effects of a less intensive and less complex intervention for children with mild brain injury, found an effect on symptoms reported on the HBI, with very large effect sizes. We are not aware of pediatric studies that are directly comparable to the current study.

#### Process evaluation

A process evaluation of the intervention is planned, based on information from the children, parents, teachers, and therapists. A combination of quantitative and qualitative data will be used, in accordance with the recommendations provided by the Medical Research Council [83]. The participation rate, number of consultations, length of sessions and preparation time, completion of the intervention in accordance with protocol, and reasons for non-compliance will be assessed as a part of the process evaluation. Approximately 10% of family sessions will be overseen by a senior researcher to evaluate treatment fidelity.

#### Dissemination plans

We aim to publish results from the study in international peer-reviewed journals of neuropsychology, neurology, pediatrics, and brain injury rehabilitation. Experiences with the study and its results will also be disseminated in relevant expert forums, national and international meetings, conferences, popular scientific journals, and reports. The results will also be shared with the user organization and its members through their communication channels in print and on the Internet/social media.

## Discussion

The intervention targets the specific areas that are challenging for children and their families in everyday life. It is a complex and innovative RCT that combines an individualized goal-oriented approach with parenting groups and school sessions, in line with current recommendations in pABI research [43]. Although many studies include only children with TBI, chronic symptoms of ABI largely overlap with TBI symptoms and are highly heterogeneous and in equal need of rehabilitation. We have thus chosen to include children with mixed etiologies.

As far as we know, this innovative RCT is the first of its kind for several reasons. To our knowledge, it is the first study to examine the effects of a comprehensive individualized and goal-oriented intervention for children in the chronic stage of ABI using a robust study design and standardized outcome measures. The study includes not only children with ABI, but also parents and schools. The combination of family and school intervention may contribute to a shared understanding of the child’s strengths and problems and enable parents and teachers to work together towards specific goals. The intervention is also expected to raise awareness of educators about the effects of pABI on learning and behavior and may increase the rate of special education services among the participants. The intensity of the intervention, with twelve sessions during approximately 6 months, enables close monitoring of the strategies and swift adjustments when necessary. In combination with the high degree of patient involvement, the close monitoring and swift adjustments are expected to lead to a feeling of control and self-efficacy in the participating children, parents, and educators. Moreover, the individualized and goal-oriented approach may provide participants with problem-solving strategies that they could continue to apply on everyday challenges after the end of the intervention, heightening the possibility of long-term effects. By also inviting local health care providers to attend school sessions where information about pABI is provided, the intervention may contribute to an increased knowledge transfer to primary health care, in addition to schools.

The RCT design allows us to establish if the intervention is effective shortly after treatment and at a 9-month follow-up. As the intervention is individually tailored, it might be applicable to other populations with neurological deficits or neurodevelopmental disorders, such as patients with cerebral palsy and ADHD. Furthermore, the study will provide insight into the use of videoconference in an intervention with children, families, and schools. Users will be involved throughout the project period, providing contributions from a first-person perspective. Finally, children with pABI are a considerable public health burden and effective rehabilitation might offer considerable socioeconomic gains, as well as increased quality of life for families. Provided effective, the treatment delivery form and its content may provide a model for future services to the pABI population. Given that the study is provided at the largest rehabilitation facility in Norway, implementation of study findings will be highly feasible.

### Limitations

The CICI study is a pragmatic clinical trial; thus, blinding of participants and therapists is not possible. However, outcome assessors will be blinded. Participants might not be representative of the whole of Norway. However, we recruit from multiple centers in the most densely populated part of Norway, which increases external validity. Also, participants from other parts of the country might be included at a later stage if recruitment is slow. Due to the limited number of available participants and the limited timeframe, the sample size is not considered very large, yet this will be one of the largest studies of rehabilitation after pABI worldwide. Also due to the limited number of participants, a vast age range is included, possibly concealing age effects.

The use of videoconference as the main channel of communication between therapists and families may prove challenging regarding optimally active involvement of the children and adolescents. It is possible that slight delays in picture and sound and the lack of rich stimuli will make it more difficult for those with existing communication or sensory integration problems to interact optimally with the therapists. On the other hand, not having to travel to the hospital for sessions will enable more families to participate and is assumed to be highly beneficial for children and adolescents with fatigue, which will affect a substantial proportion of the sample. Also, there is already some evidence that telerehabilitation may work with children [84, 85], even though more research is needed. Telerehabilitation in general can contribute to a more equally distributed service delivery, irrespective of geography, and reduced costs by reducing travel time for therapists. Without telehealth, the extensive number of sessions in this study would require more than three therapists. Furthermore, the use of telerehabilitation will enable administration of sessions despite the COVID-19 pandemic.

The intervention is individualized, which enables tailoring to specific pABI consequences for each family, increasing the effect yet possibly decreasing comparability of interventions across participants. However, the use of standard tools to measure changes across several domains will facilitate comparisons across participants. It may nonetheless be difficult to isolate the active treatment ingredients given the multipronged focus of the intervention. Despite possible limitations, an individualized, complex intervention seems to be the most sensible approach for this population, as the consequences of pABI are highly complex and affect children, their families, and schools in various ways depending on their particular context and the interplay of a vast array of biopsychosocial factors.

## Trial status

Protocol version 3.0, protocol date March 15, 2021. Recruitment for the RCT started in April 2021 and will continue until the target sample size is reached, which is expected to occur during the spring of 2023.

## Supplementary Information

**Additional file 1.** SPIRIT checklist

**Additional file 2.** Ethical approval document (translated)

**Additional file 3.** Funding documentation (translated)

## Data Availability

Not applicable.

## Abbreviations

CICI: Child in Context Intervention
ABI: Acquired brain injury
pABI: Pediatric acquired brain injury
TBI: Traumatic brain injury
RCT: Randomized controlled trial
GAS: Goal attainment scaling
CT: Computed tomography
MRI: Magnetic resonance imaging
SPIRIT: Standard Protocol Items Recommendations for Interventional Trials
HBI: Health and Behavior Inventory
TOPSE: Tool to measure Parenting Self-Efficacy
BRIEF-2: Behavior Rating Inventory of Executive Function 2
PedsQL: Pediatric Quality of Life Inventory
FNQ-P: Family Needs Questionnaire Pediatric Version
PHQ-9: Patient Health Questionnaire-9
HAD-7: A brief measure for assessing generalized anxiety disorder
WISC-V: Wechsler Intelligence Scale for Children, 5th edition

## References

[1] Anderson V, Catroppa C, Morse S, Haritou F, Rosenfeld J (2005). Functional plasticity or vulnerability after early brain injury. Pediatrics..

[2] McKinlay A, Grace RC, Horwood LJ, Fergusson DM, Ridder EM, MacFarlane MR (2008). Prevalence of traumatic brain injury among children, adolescents and young adults: prospective evidence from a birth cohort. Brain Injury..

[3] Babikian T, Merkley T, Savage RC, Giza CC, Levin H (2015). Chronic aspects of pediatric traumatic brain injury: review of the literature. J Neurotrauma..

[4] Rosema S, Crowe L, Anderson V (2012). Social function in children and adolescents after traumatic brain injury: a systematic review 1989-2011. J Neurotrauma..

[5] Slomine B, Locascio G (2009). Cognitive rehabilitation for children with acquired brain injury. Dev Disabil Res Rev..

[6] Crowe LM, Catroppa C, Anderson V (2015). Sequelae in children: developmental consequences. Handb Clin Neurol..

[7] Anderson V, Moore C (1995). Age at injury as a predictor of outcome following pediatric head injury: a longitudinal perspective. Child Neuropsychol..

[8] Catroppa C, Anderson V, Beauchamp MH, Yeates KO. New frontiers in pediatric traumatic brain injury: an evidence base for clinical practice. New York: Routledge; 2015.

[9] Heinemann AW, Sokol K, Garvin L, Bode RK (2002). Measuring unmet needs and services among persons with traumatic brain injury. Arch Phys Med Rehabil..

[10] Bruce SS-GL, Savage R (2004). Strategies for managing challenging behaviors of students with brain injuries.

[11] Wade SL, Kurowski BG (2017). Behavioral clinical trials in moderate to severe pediatric traumatic brain injury: challenges, potential solutions, and lessons learned. J Head Trauma Rehabil..

[12] Fuentes MM, Wang J, Haarbauer-Krupa J, Yeates KO, Durbin D, Zonfrillo MR, et al. Unmet Rehabilitation Needs After Hospitalization for Traumatic Brain Injury. Pediatrics. 2018;141(5):e20172859. 10.1542/peds.2017-2859. 10.1542/peds.2017-2859 PMC5914497 29674358

[13] Keetley R, Radford K, Manning JC (2019). A scoping review of the needs of children and young people with acquired brain injuries and their families. Brain Inj..

[14] Yeates KO, Taylor HG, Walz NC, Stancin T, Wade SL (2010). The family environment as a moderator of psychosocial outcomes following traumatic brain injury in young children. Neuropsychology..

[15] Yeates KO, Taylor HG, Drotar D, Wade SL, Klein S, Stancin T (1997). Preinjury family environment as a determinant of recovery from traumatic brain injuries in school-age children. J Int Neuropsychol Soc..

[16] Tuerk C, Gagner C, Dégeilh F, Bellerose J, Lalonde G, Landry-Roy C, Séguin M, de Beaumont L, Gravel J, Bernier A, Beauchamp MH (2020). Quality of life 6 and 18 months after mild traumatic brain injury in early childhood: an exploratory study of the role of genetic, environmental, injury, and child factors. Brain Res..

[17] Gerring JP, Wade S (2012). The essential role of psychosocial risk and protective factors in pediatric traumatic brain injury research. J Neurotrauma..

[18] Glang A, Ettel D, Todis B, Gordon WA, Oswald JM, Vaughn SL, Connors SH, Brown M (2015). Services and supports for students with traumatic brain injury: survey of state educational agencies. Exceptionality..

[19] Todis B, Glang A, Bullis M, Ettel D, Hood D (2011). Longitudinal investigation of the post-high school transition experiences of adolescents with traumatic brain injury. J Head Trauma Rehabil..

[20] Glang A, Todis B, Ettel D, Wade SL, Yeates KO (2018). Results from a randomized trial evaluating a hospital-school transition support model for students hospitalized with traumatic brain injury. Brain Inj..

[21] Linden MA, Braiden HJ, Miller S (2013). Educational professionals’ understanding of childhood traumatic brain injury. Brain Inj..

[22] Roscigno CI, Fleig DK, Knafl KA (2015). Parent management of the school reintegration needs of children and youth following moderate or severe traumatic brain injury. Disabil Rehabil..

[23] Kingery KM, Narad ME, Taylor HG, Yeates KO, Stancin T, Wade SL (2017). Do children who sustain traumatic brain injury in early childhood need and receive academic services 7 years after injury?. J Dev Behav Pediatr..

[24] Dettmer J, Ettel D, Glang A, McAvoy K (2014). Building statewide infrastructure for effective educational services for students with TBI: promising practices and recommendations. J Head Trauma Rehabil..

[25] Haarbauer-Krupa, J, Glang, A, Kurowski, B, Breiding, MJ. Report to Congress: the management of traumatic brain injury in children. Atlanta: National Center for Injury Prevention and Control (U.S.), Division of Unintentional Injury Prevention; Centers for Disease Control and Prevention (U.S.); 2018 [cited on 1 August 2021]. Available from: https://stacks.cdc.gov/view/cdc/51852.

[26] Catroppa C, Anderson VA, Muscara F, Morse SA, Haritou F, Rosenfeld JV, Heinrich LM (2009). Educational skills: long-term outcome and predictors following paediatric traumatic brain injury. Neuropsychol Rehabil..

[27] Schrieff-Elson LETKGF, Rohlwink UK, PD DRC, Rutka J, Pediatric traumatic brain injury: outcomes and rehabilitation (2017). Textbook of pediatric neurosurgery.

[28] Limond J, Leeke R (2005). Practitioner review: cognitive rehabilitation for children with acquired brain injury. J Child Psychol Psychiatry..

[29] Laatsch L, Harrington D, Hotz G, Marcantuono J, Mozzoni MP, Walsh V, Hersey KP (2007). An evidence-based review of cognitive and behavioral rehabilitation treatment studies in children with acquired brain injury. J Head Trauma Rehabil..

[30] Catroppa C, Soo C, Crowe L, Woods D, Anderson V (2012). Evidence-based approaches to the management of cognitive and behavioral impairments following pediatric brain injury. Future Neurol..

[31] Laatsch L, Dodd J, Brown T, Ciccia A, Connor F, Davis K, Doherty M, Linden M, Locascio G, Lundine J, Murphy S, Nagele D, Niemeier J, Politis A, Rode C, Slomine B, Smetana R, Yaeger L (2020). Evidence-based systematic review of cognitive rehabilitation, emotional, and family treatment studies for children with acquired brain injury literature: From 2006 to 2017. Neuropsychol Rehabil..

[32] Resch C, Rosema S, Hurks P, de Kloet A, van Heugten C (2018). Searching for effective components of cognitive rehabilitation for children and adolescents with acquired brain injury: a systematic review. Brain Injury..

[33] Wade SL, Narad ME, Shultz EL, Kurowski BG, Miley AE, Aguilar JM, Adlam AR (2018). Technology-assisted rehabilitation interventions following pediatric brain injury. J Neurosurg Sci..

[34] Ross KA, Dorris L, McMillan T (2011). A systematic review of psychological interventions to alleviate cognitive and psychosocial problems in children with acquired brain injury. Dev Med Child Neurol..

[35] Backeljauw B, Kurowski BG (2014). Interventions for attention problems after pediatric traumatic brain injury: what is the evidence. PM R.

[36] Wade SL, Michaud L, Brown TM (2006). Putting the pieces together: preliminary efficacy of a family problem-solving intervention for children with traumatic brain injury. J Head Trauma Rehabil..

[37] Wade SL, Walz NC, Carey J, McMullen KM, Cass J, Mark E (2011). Effect on behavior problems of teen online problem-solving for adolescent traumatic brain injury. Pediatrics..

[38] Wade SL, Carey J, Wolfe CR (2006). An online family intervention to reduce parental distress following pediatric brain injury. J Consult Clin Psychol..

[39] Narad ME, Minich N, Taylor HG, Kirkwood MW, Brown TM, Stancin T, Wade SL (2015). Effects of a Web-based intervention on family functioning following pediatric traumatic brain injury. J Dev Behav Pediatr..

[40] Raj SP, Wade SL, Cassedy A, Taylor HG, Stancin T, Brown TM, Kirkwood MW (2014). Parent psychological functioning and communication predict externalizing behavior problems after pediatric traumatic brain injury. J Pediatr Psychol..

[41] Wade SL, Kurowski BG, Kirkwood MW, Zhang N, Cassedy A, Brown TM, Nielsen B, Stancin T, Taylor HG (2015). Online problem-solving therapy after traumatic brain injury: a randomized controlled trial. Pediatrics..

[42] Braga LW, Da Paz AC, Ylvisaker M (2005). Direct clinician-delivered versus indirect family-supported rehabilitation of children with traumatic brain injury: a randomized controlled trial. Brain Injury..

[43] Wade DT (2020). What is rehabilitation? An empirical investigation leading to an evidence-based description. Clin Rehabil..

[44] Borgen IMH, Løvstad M, Andelic N, Hauger S, Sigurdardottir S, Søberg HL, Sveen U, Forslund MV, Kleffelgård I, Lindstad MØ, Winter L, Røe C (2020). Traumatic brain injury-needs and treatment options in the chronic phase: study protocol for a randomized controlled community-based intervention. Trials..

[45] Winter L, Moriarty HJ, Robinson K, Piersol CV, Vause-Earland T, Newhart B, Iacovone DB, Hodgson N, Gitlin LN (2016). Efficacy and acceptability of a home-based, family-inclusive intervention for veterans with TBI: a randomized controlled trial. Brain Inj..

[46] Brennan D, Tindall L, Theodoros D, Brown J, Campbell M, Christiana D, Smith D, Cason J, Lee A (2010). A blueprint for telerehabilitation guidelines. Int J Telerehabil..

[47] McCarron RH, Watson S, Gracey F (2019). What do kids with acquired brain injury want? Mapping neuropsychological rehabilitation goals to the international classification of functioning, disability and health. J Int Neuropsychol Soc..

[48] Locke EA, Latham GP (2002). Building a practically useful theory of goal setting and task motivation. A 35-year odyssey. Am Psychol..

[49] Gagnon A, Lin J, Stergiou-Kita M (2016). Family members facilitating community re-integration and return to productivity following traumatic brain injury - motivations, roles and challenges. Disabil Rehabil..

[50] Chan A-W, Tetzlaff JM, Gøtzsche PC, Altman DG, Mann H, Berlin JA (2013). SPIRIT 2013 explanation and elaboration: guidance for protocols of clinical trials. BMJ.

[51] Wechsler D (2014). Wechler Intelligence Scale for Children.

[52] Gioia GA, Isquit PK, Guy SC, Kenworthy L (2015). BRIEF-2: Behavior Rating Inventory of Executive Function.

[53] Varni JW, Seid M, Kurtin PS (2001). PedsQL (TM) 4.0: reliability and validity of the pediatric quality of life Inventory (TM) Version 4.0 generic core scales in healthy and patient populations. Med Care..

[54] Ayr LK, Yeates KO, Taylor HG, Browne M (2009). Dimensions of postconcussive symptoms in children with mild traumatic brain injuries. J Int Neuropsychol Soc..

[55] Kroenke K, Spitzer RL, Williams JBW (2001). The PHQ-9 - validity of a brief depression severity measure. J Gen Intern Med..

[56] Spitzer RL, Kroenke K, Williams JBW, Lowe B (2006). A brief measure for assessing generalized anxiety disorder - the GAD-7. Arch Intern Med..

[57] Kendall S, Bloomfield L (2005). Developing and validating a tool to measure parenting self-efficacy. J Adv Nurs..

[58] Epstein N, Baldwin L, Bishop DS (1983). The McMaster family assessment device. J Marital Fam Ther..

[59] Gan C, Wright FV (2019). Development of the family needs questionnaire - pediatric version [FNQ-P] - phase I. Brain Inj..

[60] Craig P, Dieppe P, Macintyre S, Michie S, Nazareth I, Petticrew M (2008). Developing and evaluating complex interventions: the new Medical Research Council guidance. BMJ..

[61] Skivington K, Matthews L, Simpson SA, Craig P, Baird J, Blazeby JM, et al. A new framework for developing and evaluating complex interventions: Update of Medical Research Council guidance. BMJ. 2021;374:n2061. 10.1136/bmj.n2061. 10.1136/bmj.n2061 PMC8482308 34593508

[62] Holthe IL, Rohrer-Baumgartner N, Svendsen EJ, Hauger SL, Forslund MV, IMH B, et al. Treating chronic symptoms of pediatric acquired brain injury - feasibility and acceptability of a complex telerehabilitation intervention: the Child in Context Intervention Study (CICI). Manuscr Prep. 2021. 10.3390/jcm11092564 PMC9103299 35566690

[63] Bovend'Eerdt TJ, Botell RE, Wade DT (2009). Writing SMART rehabilitation goals and achieving goal attainment scaling: a practical guide. Clin Rehabil..

[64] Malec JF (1999). Goal attainment scaling in rehabilitation. Neuropsychol Rehabil..

[65] Turner-Stokes L (2009). Goal attainment scaling (GAS) in rehabilitation: a practical guide. Clin Rehabil..

[66] Sohlberg MM (2012). Cognitive rehabilitation manual: translating evidence-based recommendations into practice. Arch Clin Neuropsych..

[67] Eccleston C, Fisher E, Law E, Bartlett J, Palermo TM (2015). Psychological interventions for parents of children and adolescents with chronic illness. Cochrane Database Syst Rev..

[68] Antonini TN, Raj SP, Oberjohn KS, Cassedy A, Makoroff KL, Fouladi M, Wade SL (2014). A pilot randomized trial of an online parenting skills program for pediatric traumatic brain injury: improvements in parenting and child behavior. Behav Ther..

[69] Law E, Fisher E, Eccleston C, Palermo TM. Psychological interventions for parents of children and adolescents with chronic illness. Cochrane Database Syst Rev. 2019;3(6). 10.1002/14651858.CD009660.pub4. 10.1002/14651858.CD009660.pub4 PMC6450193 30883665

[70] Wade SL, Cassedy AE, Shultz EL, Zang H, Zhang N, Kirkwood MW, Stancin T, Yeates KO, Taylor HG (2017). Randomized clinical trial of online parent training for behavior problems after early brain injury. J Am Acad Child Adolesc Psychiatry..

[71] Wade SL, Carey J, Wolfe CR (2006). The efficacy of an online cognitive-behavioral family intervention in improving child behavior and social competence following pediatric brain injury. Am Psychol Assoc.

[72] Wade SL, Stancin T, Kirkwood M, Brown TM, McMullen KM, Taylor HG (2014). Counselor-assisted problem solving (CAPS) improves behavioral outcomes in older adolescents with complicated mild to severe TBI. J Head Trauma Rehabil..

[73] Dewan MC, Mummareddy N, Wellons JC, Bonfield CM (2016). Epidemiology of global pediatric traumatic brain injury: qualitative review. World Neurosurg.

[74] Dahl HM, Andelic N, Lovstad M, Holthe IL, Hestnes M, Diseth TH (2021). Epidemiology of traumatic brain injury in children 15 years and younger in South-Eastern Norway in 2015-16. Implications for prevention and follow-up needs. EJPN.

[75] Kreftregisteret (2020). Nasjonalt kvalitetsregister for barnekreft, Årsrapport 2019.

[76] Thompson C, Kneen R, Riordan A, Kelly D, Pollard AJ (2012). Encephalitis in children. Arch Dis Childhood..

[77] Sejvar J. Neuroepidemiology and the epidemiology of viral infections of the nervous system. Handb Clin Neurol. 2014;123:67–87. 10.1016/B978-0-444-53488-0.00003-1. 10.1016/B978-0-444-53488-0.00003-1 PMC4732278 25015481

[78] Ferriero DM, Fullerton HJ, Bernard TJ, Billinghurst L, Daniels SR, DeBaun MR, deVeber G, Ichord RN, Jordan LC, Massicotte P, Meldau J, Roach ES, Smith ER, American Heart Association Stroke Council and Council on Cardiovascular and Stroke Nursing (2019). Management of stroke in neonates and children: a scientific statement from the American Heart Association/American Stroke Association. Stroke..

[79] Slomine BS, McCarthy ML, Ding R, MacKenzie EJ, Jaffe KM, Aitken ME (2006). Health care utilization and needs after pediatric traumatic brain injury. Pediatrics..

[80] Jones S, Davis N, Tyson SF (2018). A scoping review of the needs of children and other family members after a child’s traumatic injury. Clin Rehabil..

[81] Faul F, Erdfelder E, Lang A-G, Buchner A (2007). G*Power 3: a flexible statistical power analysis program for the social, behavioral, and biomedical sciences. Behav Res Methods..

[82] Renaud MI, van de Port IGL, Catsman-Berrevoets CE, Köhler S, Lambregts SAM, van Heugten CM (2020). Effectiveness of the Brains Ahead! Intervention: 6 months results of a randomized controlled trial in school-aged children with mild traumatic brain injury. J Head Trauma Rehabil.

[83] Moore GF, Audrey S, Barker M, Bond L, Bonell C, Hardeman W, Moore L, O'Cathain A, Tinati T, Wight D, Baird J (2015). Process evaluation of complex interventions: Medical Research Council guidance. BMJ..

[84] Krasovsky T, Silberg T, Barak S, Eisenstein E, Erez N, Feldman I, Guttman D, Liber P, Patael SZ, Sarna H, Sadeh Y, Steinberg P, Landa J (2021). Transition to multidisciplinary pediatric telerehabilitation during the COVID-19 pandemic: strategy development and implementation. Int J Environ Res Public Health..

[85] Camden C, Pratte G, Fallon F, Couture M, Berbari J, Tousignant M (2020). Diversity of practices in telerehabilitation for children with disabilities and effective intervention characteristics: results from a systematic review. Disabil Rehabil..

[86] Association WM (2013). World Medical Association Declaration of Helsinki: ethical principles for medical research involving human subjects. Jama..

