# Supplementary material for: Rehabilitation for children with chronic acquired brain injury in the Child in Context Intervention (CICI) study: study protocol for a randomized controlled trial
Source: Trials. 2022 Feb 22;23:169. doi: 10.1186/s13063-022-06048-8 (PMC8861614; doi:10.1186/s13063-022-06048-8)
Supplement: Supplementary file 2 — Additional file 2. Ethical approval document (translated) [file 13063_2022_6048_MOESM2_ESM.pdf]

REK
REGIONALE KOMITEER FOR MEDISINSK OG HELSEFAGLIG FORSKNINGSETIKK

The Project Marianne Løvstad

**Regional Committee for Medical & Health Research Ethics**
**South East Norway, Section C**
Postbox 1130 Blindern
NO-0318 Oslo
Norway

Phone: + 47 22 84 55 98
E-mail: t.t.mosling@medisin.uio.no
Webportal: http://helseforskning.etikkom.no

Our ref.: 2019/1283C

Date: 19st of November 2021

To whom it may concern,

**Re: REC Letter of Confirmation**

I am writing in reference to a request from Nina Rohrer-Baumgartner, regarding a Letter of Confirmation in English.

Confirmation
We hereby confirm that Regional Committee for Medical & Health Research Ethics, Section C, South East Norway, approved the Research Project: "The child in context: treatment of chronic sy mptoms after acquired brain injury in children. A randomized controlled trial. (Norwegian title: "Barnet i kontekst: behandling av kroniske symptomer etter ervervet hjerneskade hos barn. En randomisert kontrollert studie.") at its Committee Review Meeting on the 22th of August 2019. The Project Manager for the study is Marianne Løvstad, and the Institution Responsible for Research is Sunnaas sykehus HF.

The approval has been given on the basis that Research Project will be implemented as described in the Research Protocol.

Ethics Committee System
The Ethics Committee System in Norway consists of seven Independent Regional Committees with authority to either approve or disapprove Medical Research Studies conducted within Norway, or by Norwegian Institutions, in accordance with the Act on Medical and Health Research (2008).

Please do not hesitate to contact the Regional Committee for Medical and Health Research Ethics Section South East C (REK Sør-Øst C) if you have any questions, as we are happy to help.

Yours faithfully,

Erik Fosse
Chair of the Regional Committee for Medical
& Health Research Ethics of South East Norway,
Section C

Tone Transeth Mosling
Executive Officer
