# Supplementary material for: Rehabilitation for children with chronic acquired brain injury in the Child in Context Intervention (CICI) study: study protocol for a randomized controlled trial
Source: Trials. 2022 Feb 22;23:169. doi: 10.1186/s13063-022-06048-8 (PMC8861614; doi:10.1186/s13063-022-06048-8)
Supplement: Supplementary file 3 — Additional file 3. Funding documentation (translated) [file 13063_2022_6048_MOESM3_ESM.pdf]

The Research Council of Norway

Marianne Løvstad
SUNNAAS SYKEHUS HF
1453 Bjørnemyr

**Contact person/tel.**	**Our ref. 288172**	**Oslo, 10.12.2021**
Simona Grasso		
+47 46378332	**Your ref.**	

Confirmation of project funding

To whom it may concern,

The Research Council of Norway (RCN) hereby confirms funding the project "***The Child-In-Context-Intervention (CICI): A randomized controlled trial addressing chronic symptoms of Pediatric Acquired Brain Injury***" with project number **288172.** The program board for BEHANDLING has allocated funding for 14 999 000 NOK for the project, as decided at its meeting of 26.11.2018.

The Research Council of Norway is public government agency funding research and innovation projects across disciplines and on behalf of the Government. Our aim is to promote a society where research is created, used and shared, and thus contributes to restructuring and enhanced sustainability

For further information you may contact Simona Grasso at sgr@forskningsradet.no

Yours sincerely,
The Research Council of Norway

**Ole Johan Borge**	**Simona Grasso**
Head of the Department	Senior adviser
Division for health research and innovation	Division for health research and innovation

This letter has been approved and expedited electronically
